# The anaplerotic node is essential for the intracellular survival of *Mycobacterium tuberculosis*

**DOI:** 10.1074/jbc.RA118.001839

**Published:** 2018-02-23

**Authors:** Piyali Basu, Noor Sandhu, Apoorva Bhatt, Albel Singh, Ricardo Balhana, Irene Gobe, Nicola A. Crowhurst, Tom A. Mendum, Liang Gao, Jane L. Ward, Michael H. Beale, Johnjoe McFadden, Dany J. V. Beste

**Affiliations:** From the ‡ Department of Microbial and Cellular Sciences, Faculty of Health and Medical Sciences, University of Surrey, Guildford GU2 7XH,; the § School of Biosciences, University of Birmingham, Edgbaston, Birmingham B15 2TT, and; the ¶ Department of Computational and Analytical Sciences, Rothamsted Research, Harpenden, Herts AL5 2JQ, United Kingdom

**Keywords:** tuberculosis, microbial pathogenesis, microbiology, host-pathogen interaction, Mycobacterium tuberculosis, gluconeogenesis, enzyme, Microbial metabolism

## Abstract

Enzymes at the phosphoenolpyruvate (PEP)–pyruvate–oxaloacetate or anaplerotic (ANA) node control the metabolic flux to glycolysis, gluconeogenesis, and anaplerosis. Here we used genetic, biochemical, and ^13^C isotopomer analysis to characterize the role of the enzymes at the ANA node in intracellular survival of the world's most successful bacterial pathogen, *Mycobacterium tuberculosis* (*Mtb*). We show that each of the four ANA enzymes, pyruvate carboxylase (PCA), PEP carboxykinase (PCK), malic enzyme (MEZ), and pyruvate phosphate dikinase (PPDK), performs a unique and essential metabolic function during the intracellular survival of *Mtb.* We show that in addition to PCK, intracellular *Mtb* requires PPDK as an alternative gateway into gluconeogenesis. Propionate and cholesterol detoxification was also identified as an essential function of PPDK revealing an unexpected role for the ANA node in the metabolism of these physiologically important intracellular substrates and highlighting this enzyme as a tuberculosis (TB)-specific drug target. We show that anaplerotic fixation of CO_2_ through the ANA node is essential for intracellular survival of *Mtb* and that *Mtb* possesses three enzymes (PCA, PCK, and MEZ) capable of fulfilling this function. In addition to providing a back-up role in anaplerosis we show that MEZ also has a role in lipid biosynthesis. MEZ knockout strains have an altered cell wall and were deficient in the initial entry into macrophages. This work reveals that the ANA node is a focal point for controlling the intracellular replication of *Mtb,* which goes beyond canonical gluconeogenesis and represents a promising target for designing novel anti-TB drugs.

## Introduction

*Mycobacterium tuberculosis* (*Mtb*)^2^ is the causative agent of a global tuberculosis epidemic that has now reached staggering levels and is the biggest infectious disease killer worldwide (1). The limited number of drugs available that have activity against *Mtb*, the lengthy multidrug regimen needed to eradicate the infection, and the worldwide spread of multi- and extensively drug-resistant strains (2, 3) all complicate the treatment of tuberculosis (TB) and thwart attempts to control this global emergency. *Mtb* is an unusual bacterial pathogen, with the remarkable ability to cause both acute life threatening disease and also clinically latent infections that can persist for the lifetime of the human host. Metabolic reprogramming in response to the host niche during both acute and chronic phases of TB infections is a crucial determinant of virulence (4, 5). Experimental evidence has identified central carbon metabolism as instrumental in this pathogenic strategy (4, 6). Therefore an in-depth knowledge and understanding of the central metabolism both *in vitro* and *in vivo* will expedite the identification and validation of novel strategies to combat TB.

We previously applied the systems-based tool ^13^C-flux spectral analysis (^13^C-FSA) to show that intracellular *Mtb* co-metabolizes multiple gluconeogenic and glycolytic carbon substrates by utilizing the reactions of the phosphoenolpyruvate (PEP)–pyruvate–oxaloacetate (OAA) or anaplerotic (ANA) node, in both the gluconeogenic and carbon-fixing anaplerotic direction simultaneously (7). The ANA node consists of several bidirectional reactions, modeling is not able to predict which genes and reactions are required nor their directionality (8). The ANA node lies at the heart of the central metabolism inter-connecting the main pathways of the central metabolism (glycolysis, gluconeogenesis, and the TCA cycle) controlling the distribution of flux between anabolism, catabolism, and energy supply to the cell (9). At this node the end products of glycolysis, PEP and pyruvate, either: 1) serve directly as precursors for anabolism or 2) enter the TCA cycle via acetyl Co-A or anaplerotic reactions and this node is also the starting point for gluconeogenesis.

**Figure 1.**
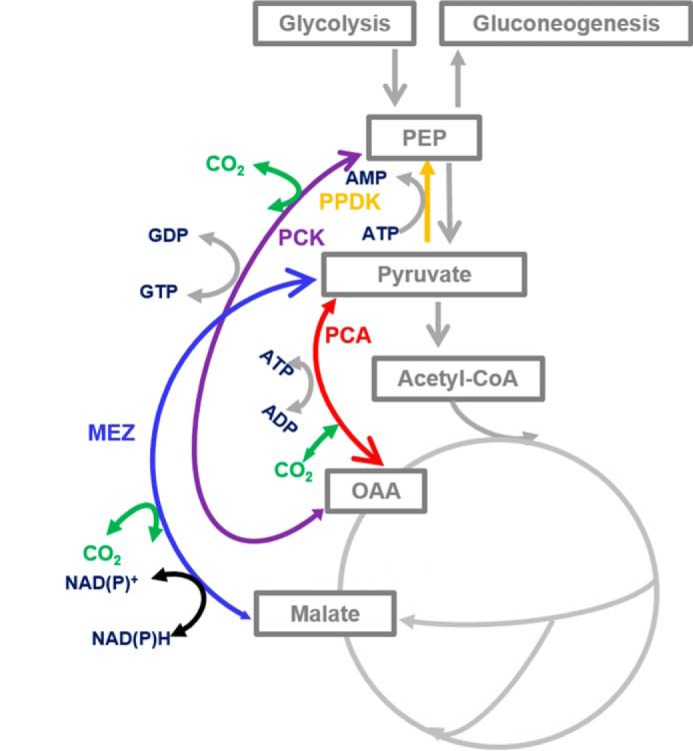
**The PEP–pyruvate–oxaloacetate node or ANA node of metabolism in *Mtb*.** The *larger arrows* point into the predicted physiological direction.

The ANA node of *Mtb* consists of the enzymes pyruvate carboxylase (PCA), PEP carboxykinase (PCK), malic enzyme (MEZ), and pyruvate phosphate dikinase (PPDK) (Fig. 1). Of these enzymes, only PCK has been shown to have an essential role in gluconeogenesis when *Mtb* is growing on lipid substrates *in vitro* and for survival in macrophage and murine models of TB (4) or hypoxia in a chemostat (10). The role of the other members of the ANA node in TB pathogenesis remains unexplored.

In this study we use a combination of genetics and biochemistry to test the redundancy within the ANA node to establish whether targeting its deregulation could inhibit intracellular nutrient acquisition and therefore intracellular growth. We show that in addition to being a gateway into both gluconeogenesis and anaplerosis, the ANA node has unexpected roles in cholesterol and propionate detoxification and lipid synthesis. Despite apparent functional overlap, we show that each enzyme of the ANA node fulfils a distinct metabolic function during the intracellular growth of *Mtb* and is thereby a potentially fruitful target for drug development.

## Results

*In silico* Genome Scale Metabolic Networks (11–13) indicate that the ANA node of *Mtb* consists of a set of functionally redundant enzymes (Fig. 1). Nevertheless, the essential role of PCK during gluconeogenesis and intracellular survival (4) demonstrates that at least this enzyme plays a role that cannot be complemented by other ANA enzymes. To define the role of each enzyme in the ANA node we generated single mutant H37Rv strains Δ*pca*, Δ*pck*, Δ*ppdk*, Δ*mez* and also a double mutant strain Δ*pca*–Δ*pck*. The growth rates of these mutants were similar to that of the WT strain when cultured in standard Middlebrook 7H9 with glycerol and Tween 80 demonstrating that these enzymes are not required for bacterial growth in rich media (Fig. S1).

### The ANA node is required for intracellular invasion and replication

**Figure 2.**
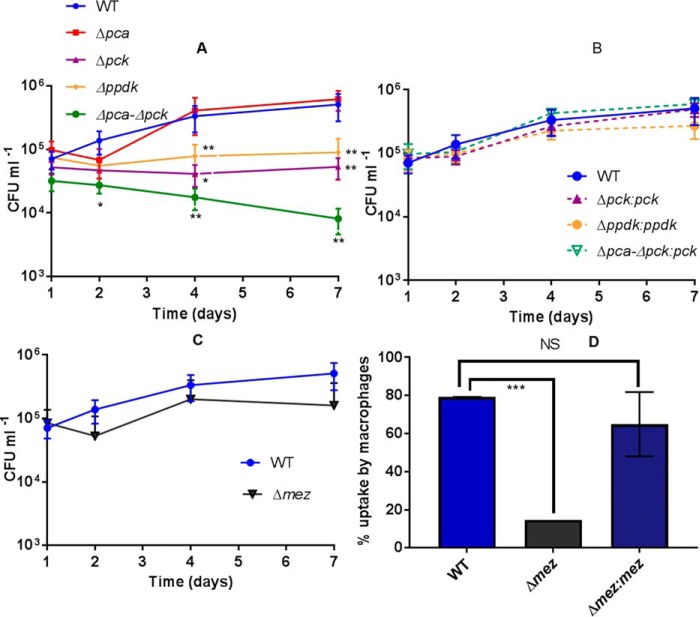
**Invasion and intracellular survival of the ANA mutants in THP-1 macrophages.** *A–D*, intracellular survival of (*A*) WT, Δ*pca*, Δ*pck*, Δp*pdk*, Δ*pca*–Δ*pck*, (*B*) respective complemented strains, (*C*) Δ*mez* and (*D*) uptake of Δ*mez* in THP-1 macrophages. Data represent the mean of 3–6 independent experiments; *error bars* indicate the S.E. *NS*, not significant; *, *p* < 0.05; **, *p* < 0.01; ***, *p* < 0.001 relative to the WT control.

To test whether the ANA node is important for *Mtb* during intracellular growth we infected THP-1 macrophages with WT, Δ*pca*, Δ*pck*, Δ*ppdk*, Δ*mez*, and Δ*pca*–Δ*pck* (Fig. 2*A*) and their respective complemented strains (Fig. 2*B*) of *Mtb* and monitored intracellular invasion and survival. Deletion of *pca* had no observable effect on intracellular bacterial growth (Fig. 2*A*). However, surprisingly deletion of both *pca* and *pck* resulted in severe impairment of intracellular survival and loss of the strain from the macrophage (Fig. 2, *A* and *B*). As described previously for the Erdman strain of *Mtb* (4) Δ*pck* H37Rv also had impaired survival but this phenotype was not as severe in comparison to Δ*pca*–Δ*pck* (Fig. 2, *A* and *B*). These results demonstrated that PCA is essential for intracellular growth in the absence of PCK.

Δ*ppdk* had a similar intracellular growth defect to *Mtb* strains lacking PCK (Fig. 2*A*) indicating that this enzyme is also performing an essential role during infection. Although intracellular Δ*mez* replicated as the parental strain this mutant was less efficient at entering macrophages indicating an invasion phenotype (Fig. 2, *C* and *D*).

### Anaplerosis through the ANA node is essential for intracellular growth

To understand the intracellular phenotypes we next investigated the ability of our ANA enzyme mutants to grow on physiologically relevant carbon sources. PCA was required for growth on pyruvate and substrates that enter metabolism as pyruvate (alanine, serine, and lactate) (Table S1). This phenotype could be complemented by reintroducing the gene into the genome under control of the constitutive *hsp*60 promoter into the *att*B site; but could also be complemented by addition of aspartate (as proxy for oxaloacetate, which is labile (14) to the media or by growing the cultures in the presence of 5% CO_2_ (Table S1). These results demonstrated that PCA is performing an anaplerotic function replenishing oxaloacetate (hence aspartate rescues the mutant) into the TCA cycle when growing on these substrates; but that this role can be complemented when sufficient CO_2_ is present presumably by the actions of either MEZ or PCK. It has already been shown by us and others that *Mtb* PCK can operate in the carboxylating (anaplerotic) direction (15, 16), however, the role of MEZ is unknown.

PCK was required for the growth of *Mtb* H37Rv on all gluconeogenic substrates tested (Tables S1 and S2) including those identified as potential intracellular carbon sources by us and others (acetate, glutamate, asparagine, cholesterol, alanine, and combinations of these substrates and their by-products) (17). This is in accordance with data for *Mtb* Erdman, which showed that the gluconeogenic flux of acetate was blocked in the absence of PCK (4). The double Δ*pca*–Δ*pck* mutant grew identically to the Δ*pck* mutant on the media tested, except that this strain was additionally unable to grow on glycerol unless supplemented with 5% CO_2_ (Table S1) suggesting that, *in vitro*, MEZ is able to complement the anaplerotic function of PCA if provided with sufficient amounts of CO_2_.

**Figure 3.**
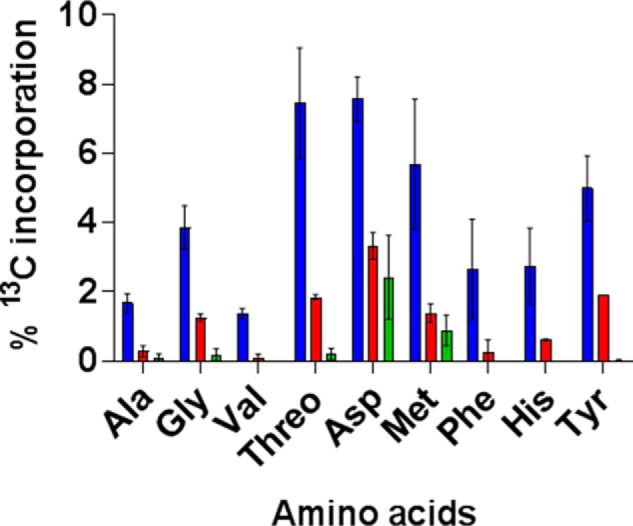
**PCK and PCA are both required for the fixation of carbon from CO_2_ when *Mtb* is growing intracellularly within THP-1 macrophages.** Shown is ^13^C incorporation into proteogenic amino acids by WT *Mtb* (*blue bars*), Δ*pca* (*red bars*), and Δ*pca*–Δ*pck* (*green bars*) after 48 h of growing intracellularly within THP-1 macrophages in the presence of sodium [^13^C]bicarbonate. *Error bars* indicate S.D. of 3–4 samples from independent macrophage infections.

To directly test the hypothesis that PCA is performing an anaplerotic function during intracellular growth and that MEZ functions anaplerotically in the absence of both PCA and PCK we infected unlabeled THP-1 macrophages with WT H37Rv, Δ*pca*, and Δ*pca*–Δ*pck* in RPMI medium containing sodium [^13^C]bicarbonate/RPMI and compared the labeling profile of amino acids after 48 h. We previously performed this experiment with Δ*pck* and showed that this enzyme was contributing to the intracellular fixation of CO_2_ (7). In the absence of PCA, ^13^C incorporation into proteogenic amino acids was significantly reduced as compared with the WT strain demonstrating that this enzyme was also contributing to the fixation of carbon from CO_2_ during intracellular growth (Fig. 3, Table S3). The results are consistent with the *in vitro* evidence that PCA functions primarily as an anaplerotic enzyme. In the double knockout of PCA and PCK ^13^C labeling of amino acids was further reduced as compared with Δ*pca* but there were still small amounts of ^13^C labeling in aspartate and methionine demonstrating that there is an additional enzyme fixing carbon into OAA, presumably MEZ (Fig. 3, Table S3). However, this diminished CO_2_ fixation correlated with reduced intracellular survival highlighting the importance of anaplerosis through the ANA node for infection.

### MEZ has a role in lipid biosynthesis

Although Δ*mez* was dispensable for the growth of *Mtb* H37Rv on all gluconeogenic substrates and glycolytic substrates tested (Tables S1 and S2), the mutant displayed a glossy and viscous morphology on solid media, which combined with the impaired invasion phenotype suggested that this strain may have an altered cell wall lipid profile.

**Figure 4.**
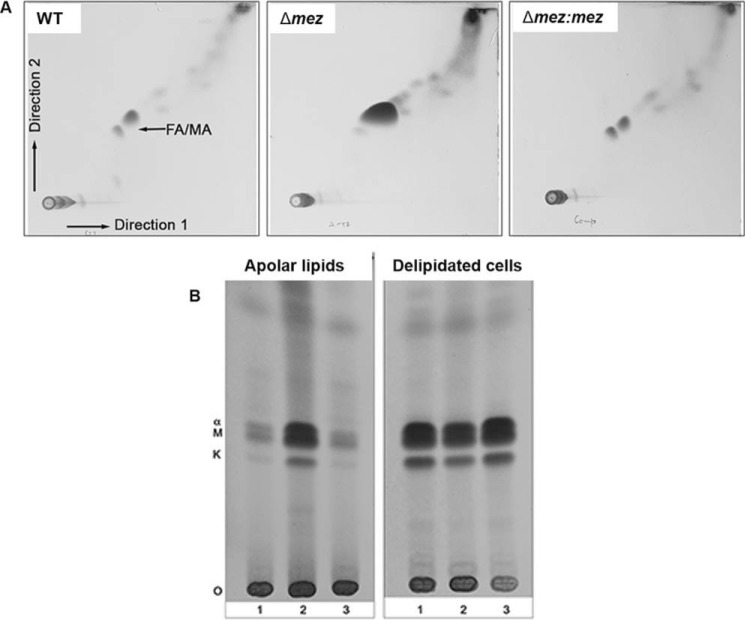
**Loss of MEZ leads to the accumulation of free mycolic acids.** *A,* TLC analysis of apolar lipids extracted from WT, Δ*mez*, and Δ*mez:mez* separated by 2D TLC using solvent system C: chloroform/methanol (96:4, v/v) in the first direction and toluene/acetone (80:20, v/v) in the second direction, 5% ethanolic molybdophosphoric acid, followed by charring. *B,* TLC analysis of MAMEs derived from apolar lipids and delipidated cells of: 1) WT; 2) Δ*mez*; and 3) Δ*mez:mez*. á, α MAMEs; *M,* methoxy MAMEs; *K,* keto MAMEs.

To test whether the altered *in vitro* growth morphology phenotype was due to alterations in the cell envelope lipid composition, we biochemically analyzed the lipid content of WT, Δ*mez*, and the complemented strain Δ*mez*: *mez.* We extracted apolar and polar lipids from the strains and analyzed the extracts by 2D thin-layer chromatography (TLC). The strains were initially treated with petroleum ether to extract outer, non-covalently bound lipids, prior to extraction of inner apolar lipids and polar lipids. The *mez* mutant showed an accumulation of a major species in solvent System C (18) that co-migrated in the same region where free fatty acids (FA) and free mycolic acids (MA) are detected (Fig. 4*A,* Fig. S2). Subsequently we extracted mycolic acid methyl esters (MAMEs) from apolar lipids (containing trehalose mono- and dimycolates, TMM and TDM, and free FA) and confirmed that Δ*mez* was accumulating free MA with an accompanying decrease in the levels of cell wall-bound mycolates from delipidated cells (Fig. 4*B*). This data were further supported by GC-MS analysis, which detected higher levels of C26 species in the Δ*mez* strain (Fig. S3). Lipid profiles were restored to those of the parental strain on complementation (Fig. 4, Figs. S2 and S3).

Malic enzymes catalyze the reversible oxidative decarboxylation of malate to produce CO_2_ and pyruvate with the concomitant reduction of NAD(P)^+^ to NAD(P)H. These enzymes vary in their reversibility and cofactor preference and their role in metabolism is often not clearly established. To validate *Mtb* MEZ functional properties we determined the catalytic properties of the purified enzyme (Table 1).

**Table 1:** **Kinetic constants for *Mtb* MEZ** Kinetic values are given as averages ± S.E.

Substrate (concentration range; pH)	*K_m_*/*K*_0.5_	*V*_max_	*V*_max_/*K_m_*
	*mm*	*mmol min*^−*1*^ μ*mol*^−*1*^	*min*^−*1*^μ*mol*^−*1*^
Gluconeogenic reaction (l-malate decarboxylation)			
l-Malate (0.25–100 mm; pH 7.4) with NAD^+^	18.86 ± 0.52*^a^*	127.3 ± 1.75	6.75
l-Malate (0.25–100 mm; pH 7.4) with NADP^+^	40.38 ± 0.51*^a^*	43.92 ± 0.39	1.09
Anaplerotic reaction (pyruvate carboxylation)			
Pyruvate (20–100 mm; pH 7.4) with NADH as cofactor	91.98 ± 6.73	5.17 ± 0.22	0.06

*^a^* Kinetics for these reactions are sigmoidal and the reported values are *K*_0.5_ values.

The enzyme was able to decarboxylate malate (forward, gluconeogenic, reaction) using either NAD^+^ or NADP^+^ with a preference for NAD^+^ demonstrating that this enzyme can provide both NADH and NADPH for lipid biosynthesis. The enzyme showed a sigmoidicity at low malate concentrations indicating homotropic allosteric regulation. The reverse reductive pyruvate carboxylation reaction showed typical Michaelis-Menten kinetics confirming that *Mtb* MEZ can serve as a backup anaplerotic enzyme for *Mtb*. However, the substrate affinities and maximum reaction rates show that the forward reaction is preferred.

### PPDK is essential for cholesterol and propionate detoxification

The combined actions of MEZ and PPDK should be able to provide *Mtb* with an alternative gluconeogenic route that circumvents the requirement for PCK (Fig. 1). Strikingly, Δ*ppdk* was able to grow on all gluconeogenic and glycolytic substrates tested with the exception of medium containing cholesterol as a sole carbon source or a combination of cholesterol and acetate, even though the mutant could grow in acetate as a sole carbon source (Tables S1 and S2). Moreover, addition of cholesterol to minimal media containing pyruvate, glycerol, or glutamate similarly inhibited the growth of Δ*ppdk* (Table S2). This phenotype has been observed in other mutant *Mtb s*trains that are unable to metabolize cholesterol and has been linked to the accumulation of toxic propionyl-CoA produced from cholesterol catabolism (19, 20).

**Figure 5.**
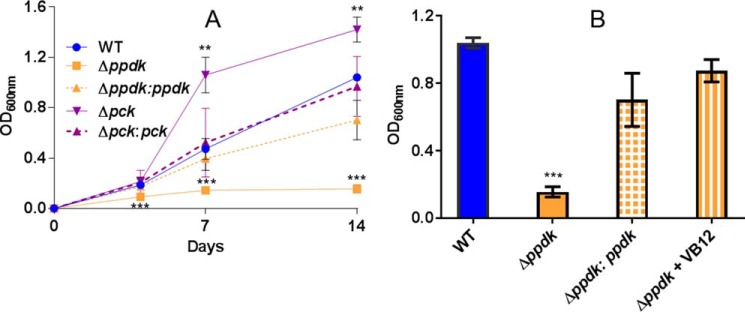
**PPDK deficiency sensitizes *Mtb* to propionate toxicity, which is rescued by the addition of vitamin B12.** *A,* Δ*ppdk* growth is inhibited in the presence of propionate. Bacterial growth measured by absorbance was determined for WT (*solid blue*), Δ*pc*k (*solid purple*), Δ*ppdk* (*solid yellow*), and Δ*ppdk:ppdk* (*dashed yellow*) grown for 14 days in 7H9 medium containing glycerol and 20 mm sodium propionate. *B,* propionate toxicity in Δ*ppdk* is rescued by the addition of 10 μg ml^−1^ of vitamin B12 (*VB12*). Bacterial growth measured by absorbance was determined after 14 days. *Error bars* are representative of S.E. of 3–6 independent biological replicates. **, *p* < 0.01; ***, *p* < 0.001 relative to the WT control.

Propionate did indeed inhibit the growth of Δ*ppdk* in standard 7H9 medium (containing glycerol and Tween 80) as compared with the WT or the complemented strain Δ*ppdk*:*ppdk* (Fig. 5*A*) demonstrating that PPDK plays a role in detoxifying propionyl-CoA. In contrast Δ*pck* was not intoxicated by either cholesterol or propionyl-CoA (Table S2, Fig. 5*A*). In fact deletion of *pck* enhanced the growth of *Mtb* in the presence of propionate as compared with the WT strain (Fig. 5*A*).

*Mtb* has two pathways for metabolizing propionyl-CoA, via the methyl citrate cycle to yield pyruvate and succinate or in the presence of sufficient vitamin B12 the methyl malonyl pathway to yield succinyl-CoA. Activation of the methyl malonyl pathway with vitamin B12 restored growth of Δ*ppdk* in propionate (Fig. 5*B*) suggesting that PPDK provides a link between the methyl citrate cycle-derived pyruvate and the rest of metabolism.

**Figure 6.**
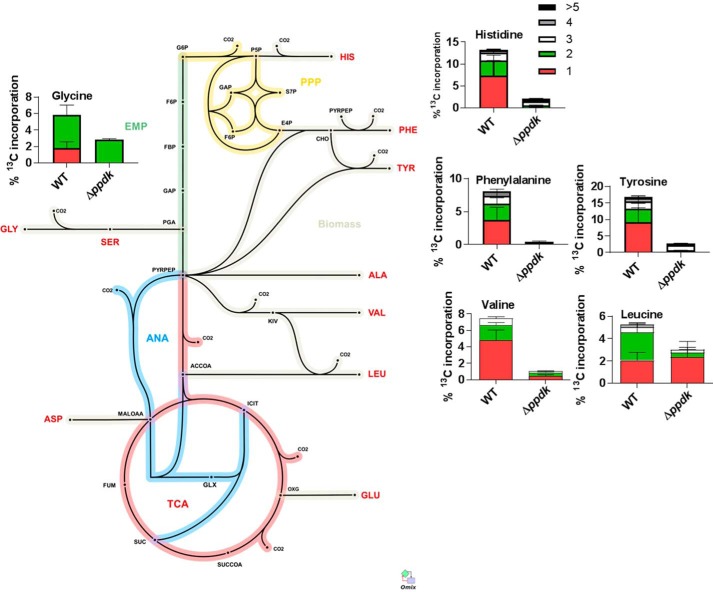
**Deletion of PPDK disrupts gluconeogenesis of intracellular *Mtb*.** Shown is _13_C isotopomer incorporation into proteogenic amino acids isolated from intracellular WT and Δ*ppdk* after 48 h infection of pre-labeled (with [U-_13_C_6_]glucose) THP-1 macrophages superimposed on a metabolic network of central metabolism with the positioning of each chart indicating the source of the carbon backbone of each amino acid. Only the amino acids that had significant changes as compared with WT are displayed. *Error bars* indicate S.D. of 3 to 4 samples from independent macrophage infections.

To further explore the intracellular metabolic profile of this mutant we performed ^13^C isotopologue experiments with prelabeled THP-1 macrophages. THP-1 macrophages were passaged in [^13^C]glucose/RPMI as previously described (7). These were infected with WT and Δ*ppdk* for 48 h. We selected this time point as the Δ*ppdk* mutant showed no detectable growth phenotype at this time so fluxes could be compared with WT (Fig. 2). Also, the labeling profile of amino acids is at an isotopic steady state by 48 h (7). These data were analyzed manually using the results from our previous flux analysis as a template (7). We focused the analysis on amino acids with ^13^C profiles, which were significantly different from the WT to identify major defects in metabolism consequent to enzyme deletion (Table S4, Fig. 6).

In the absence of PPDK there was a block in gluconeogenic flow (Table S4, Fig. 6). ^13^C enrichment occurred only in amino acids derived from metabolites occurring below pyruvate but not in those amino acids derived from glycolytic intermediates (Fig. 6, Table S4). These data show that during intracellular growth PPDK provides an alternative to PCK as a gateway into gluconeogenesis and that both of these routes are required for the intracellular survival of *Mtb*.

## Discussion

A number of studies highlight the importance of individual enzymes of glycolysis, gluconeogenesis, and the TCA cycle to the virulence strategy of *Mtb*. These pathways are interconnected by four enzymes at a metabolic cross-roads known as the ANA node (9). Here we demonstrate that although there is some functional overlap, each of these enzymes fulfils a unique metabolic function during intracellular growth. In addition to its canonical role in gluconeogenesis and anaplerosis we reveal that the ANA node has unexpected roles in lipid biosynthesis and detoxifying cholesterol and propionate.

We previously demonstrated that *Mtb* is able to fix carbon from CO_2_ both *in vitro* (15) and also when growing intracellularly (7). We show here that *Mtb* has three potential routes for the replenishment of C4 intermediates catalyzed by PCK, PCA, or MEZ and that disruption of two of these routes severely compromises the intracellular survival of *Mtb* demonstrating that CO_2_ fixation plays a fundamental role in *Mtb'*s survival in its human host cell. At ambient CO_2_ levels *in vitro* PCA was the dominant anaplerotic enzyme required for growth on pyruvate but at higher CO_2_ levels this enzyme was redundant. However, deletion of both PCA and PCK from *Mtb* led to severe intracellular growth defects.

PCA enzymes are among the most efficient carboxylating enzymes, whereas all others including MEZ and PCK have poor activity under ambient CO_2_ concentrations (21). It was previously shown by us and others that PCK can also function as a carboxylase (7, 16). Here, functional analysis of *Mtb* MEZ confirms that this enzyme can also function as a carboxylating enzyme. However, for MEZ to contribute to TCA cycle replenishment, pyruvate would need to accumulate to high intracellular concentrations and therefore MEZ is likely to play a subordinate role when alternative anaplerotic routes (PCA and PCK) are operational. Moreover MEZ was unable to compensate for the loss of both PCA and PCK in the complex intracellular environment.

Malic enzymes have been studied extensively in eukaryotes. However, the role of this enzyme in pathogenic bacteria is not well understood. Potential roles include provision of: 1) pyruvate during gluconeogenesis; 2) carbon during anaplerosis; and 3) reducing power for fatty acid synthesis (22). We confirmed that *Mtb* MEZ can function anaplerotically as a carboxylase although this enzyme preferentially catalyzes the oxidative decarboxylation of malate to pyruvate. Deletion of *mez* had no effect on metabolism of glycolytic or gluconeogenic substrates. However, we detected alterations in the colonial morphology, cell wall lipid composition, and an intracellular invasion phenotype in MEZ-deficient *Mtb*. Interestingly the excess of free mycolates detected in the mutant has also been observed in mycobacterial biofilms where it is associated with the activity of an esterase (23). Although this enzyme has a preference for NAD, *Mtb* MEZ is one of a small number of malic enzymes that have duel cofactor specificity and can therefore produce both NADH and NADPH as reductants. The dogma is that the dehydrogenase reactions of the oxidative pentose phosphate pathway are the major providers of reducing power in bacteria. However, the importance of alternative NADPH-generating reactions has recently become evident (24). Malic enzymes are associated with a role in controlling lipid metabolism through the provision of NADPH in eukaryotes (22) and we hypothesize that this is also the case for *Mtb*, which is unusual in requiring both NADH and NADPH as reductants to fuel its fatty acid synthase systems, FASI and -II. Malic enzyme may be particularly important when *Mtb* is dieting on carbon sources, which have low flux through the pentose phosphate pathway. We are currently exploring this hypothesis further.

During intracellular growth *Mtb* is reliant on gluconeogenesis to metabolize a mixture of substrates including amino acids (7), fatty acids and cholesterol (17), and mutants lacking genes involved in gluconeogenesis fail to establish infections in macrophages and murine models of TB (4, 6). Gluconeogenesis is essential for the pathogen to make amino acids (serine, histidine, phenylalanine, and tyrosine) in addition to sugars such as trehalose, which are essential for cell wall biosynthesis as well as puridines to make DNA and RNA. The inability of the *pck* deletion mutant to grow *in vitro* on any gluconeogenic substrate shown here and by others (4) indicated that PCK cannot be functionally replaced by PPDK *in vitro* suggesting that this enzyme was not operative in *Mtb* (25). Here we show that this is not the case and that PPDK is essential for intracellular survival. Indeed ^13^C isotopomer analysis demonstrated that this enzyme is in fact an essential gateway into gluconeogenesis during intracellular growth. This work further confirms that gluconeogenesis is critical for intracellular growth of *Mtb* and reveals that PPDK is essential in this process.

The metabolism of host cholesterol has an essential role in TB infection (17, 26, 27) and therefore cholesterol metabolism has been highlighted as a drug target (28, 29). Here we have shown that PPDK has an essential role in the metabolism of cholesterol. We further demonstrated that strains lacking PPDK fail to grow if cholesterol is present in the medium. This form of intoxication has been described for other mutants (20, 26, 30) where it is associated with the inability to assimilate the cholesterol breakdown product propionyl-CoA and we confirmed that this is also the case here.

Propionyl-CoA metabolism is central to the adaptation of *Mtb* to growth on cholesterol (26). *Mtb* has three strategies for metabolizing propionyl-CoA via: 1) the methyl citrate cycle to produce pyruvate and succinate; 2) the methyl malonyl pathway leading to succinyl-CoA production; or 3) incorporation into cell wall lipids (19). The growth of Δ*ppdk* was inhibited by propionate even in the presence of an alternative carbon source demonstrating that propionate, or its by-products, were intoxicating this mutant. Activation of the methyl malonyl pathway with vitamin B12 rescued this phenotype, indicating that PPDK provides a link between the methyl citrate cycle and the rest of metabolism. Propionyl-CoA toxicity is not fully understood in bacteria including *Mtb* but this metabolite has been shown to be an inhibitor of several key metabolic enzymes including pyruvate dehydrogenase (PDH) (31, 32). The end product of the methyl citrate cycle is pyruvate, which could be channeled through PPDK to circumvent the requirement for PDH. Deletion of the alternative gluconeogenic route driven by PCK actually mitigates propionyl-CoA toxicity indicating that PPDK provides a uniquely essential gluconeogenic route for metabolizing propionyl-CoA-derived metabolites.

We posit that during intracellular growth when *Mtb* is co-metabolizing a mixture of gluconeogenic carbon substrates (6, 7), having two gluconeogenic gateways provides *Mtb* with a selective advantage. PPDK provides more efficient recovery of carbon from substrates that are metabolized via pyruvate such as propionyl-CoA. Through PCK a minimum of 2 mol of pyruvate are required to form 1 mol of PEP via PDH and the glyoxylate or tricarboxylic acid cycle, whereas the molar ratio is 1:1 using PPDK. However, an additional ATP is necessary to regenerate ADP from AMP when using PPDK and therefore PCK provides an energy efficient route to make sugars from lipids. Utilizing both routes provides *Mtb* as an effective strategy to minimize propionyl-CoA toxicity and maximize ATP production. The absence of PPDK orthologues in mammals should facilitate the development of gluconeogenesis inhibitors as new drugs for the treatment of tuberculosis.

In summary we show that the ANA node acts as a gateway into gluconeogenesis and anaplerosis in addition to non-canonical roles in cholesterol detoxification and lipogenesis and is essential for the intracellular life cycle of *Mtb*. Deregulating this node offers an attractive alternative to conventional antimicrobial chemotherapy.

## Experimental procedures

### Bacterial strains and growth conditions

*Escherichia coli* strain DH5α was grown in solid or liquid Luria-Bertani (LB) media. Frozen stocks of *Mtb* were cultivated using Middlebrook 7H11 agar containing 5% (v/v) oleic acid/albumin/dextrose/catalase (OADC) enrichment medium supplement (BD) and 0.5% (v/v) glycerol or Middlebrook 7H9 broth containing 0.2% (v/v) glycerol, 0.2% (v/v) Tween 80, and 5% (v/v) ADC. When selection was required, kanamycin at 20 μg ml^−1^, hygromycin at 50 μg ml^−1^, X-gal at 50 μg ml^−1^, and zeomycin at 25 μg ml^−1^ were added to the culture medium.

For the sole carbon growth experiments cultures were grown until late exponential phase (*A*_600_ = 1.0) in complete 7H9, washed once with PBS, and plated in triplicate onto Roisin's minimal agar (33) containing sole carbon sources, duel carbon sources, and multiple carbon sources. For consistency the carbon source(s) were added to attain an equivalent carbon concentration of 109 mm with the exception of acetate, which was added at 12 mm as higher amounts were toxic to *Mtb*. Duplicate plates for each substrate were incubated for up to 12 weeks. To test for any mutations that may have given false results any positives after 6 weeks were independently tested at least twice more.

Growth curves were performed in 7H9 or Roisin's minimal media. For the propionyl-CoA toxicity assays strains were grown in 7H9 media containing 0.2% (v/v) glycerol, 0.2% (v/v) Tyloxopol, and 5% (v/v) ADC and 20 mm sodium propionate. Vitamin B12 (10 μg ml^−1^) was added when indicated. Cultures were inoculated with 1% (v/v) of washed late log phase culture (*A*_600_ = 1.0). Cell growth was monitored daily.

### Genetic manipulation

The *ppdk* mutant was constructed using the strategy described by Stewart *et al.* (34). Approximately 1-kb regions flanking *ppdk* were PCR amplified and cloned on either side of the hygromycin cassette of the suicide vector pG5, which is a pSMT100 plasmid carrying a kanamycin resistance gene in addition to the *sac*B counter-selectable marker. The resulting plasmid was electroporated into *Mtb* H37Rv as described previously (35). Double crossovers were selected for on 7H11 agar supplemented with kanamycin, hygromycin, and X-gal. The *pca* gene was disrupted by single step homologous recombination using the specialized phage transduction protocol (36). The mycobacterial recombineering system was used for the construction of the single pck and the double *pca–pck* mutants as described (37). Briefly, the allelic exchange substrates were constructed by PCR amplification of ∼500 bp corresponding to regions upstream and downstream of the gene and subsequently inserted into pNCMT so that they flank an antibiotic resistance gene, hygromycin was used as the antibiotic marker for Δpck, and zeomycin was used to generate the Δ*pca*:Δ*pck* mutant. The allelic exchange substrates were then electroporated into *Mtb* strains expressing phage recombinases. To restore the phenotypes the WT genes were reintroduced into the mutant strains using suicide vectors that insert into the *att*B site of *Mtb*. Using operon prediction software available online, *pck* was predicted not to be in an operon. *ppdk* was predicted to be the first gene in its operon therefore these genes and 500 bp upstream of the CDS were reintroduced back into *Mtb* using the plasmid pMV306. *pca* was predicted to be within an operon, and therefore this gene was cloned downstream of the *hsp*60 promoter and reintroduced into the genome using the suicide vector pMV361 PCR. Whole genome sequencing was performed to verify the expected genotypes for all mutants.

### Expression and purification of recombinant MEZ

MEZ was cloned into pNIC28-BSA4, a vector for inducible expression of His-tagged MEZ in *E. coli* using the primers 5′-TACTTCCAATCCATGAGCGACGCCCGCGT and 3′-TATCCACCTTTACTGTTAGTCATATGCCGGGAG to generate the pNIC_MEZ overexpression plasmid. The pNIC_MEZ overexpression plasmid was confirmed by Sanger sequencing and transformed into *E. coli* Tuner cells (Novagen) for overexpression in Magic Media^TM^ (ThermoFisher Scientific) containing 50 μg ml^−1^ of kanamycin at 37 °C overnight. The supernatant containing soluble protein was isolated using a nickel affinity-based His-trap column (GE Healthcare). Unbound protein was removed by washing with 15 CV of lysis buffer (50 mm Tris, pH 7.6, 500 mm NaCl, 0.1 mm β-mercaptoethanol, 5 mm MgCl_2_, 0.5 mm CaCl_2_, 2% glycerol, 0.1% Triton X-100, 25 mm imidazole) before the protein was eluted in 45 mm Tris, pH 7.6, 50 mm NaCl, 5 mm MgCl_2_, 0.5 mm CaCl_2_, 4% glycerol, 0.1% Triton X-100, 250 mm imidazole. The protein was dialyzed against 50 mm Tris, pH 7.6, 100 mm NaCl, 5 mm MgCl_2_, 0.5 mm CaCl_2_, 4% glycerol for 4 h at 4 °C, and then stored at −80 °C.

MEZ enzyme activity was measured at 30 °C by spectrophotometrically measuring the NAD(P)H formation or utilization at 340 nm. Assaying in the direction of pyruvate formation the reaction mixture contained: 100 mm Tris, pH 7.4, 100 mm malate, 0.5 mm MnCl_2_, 1.6 mm NAD(P). Each reaction mixture used to assay for pyruvate reductive carboxylating activity contained 100 mm Tris, pH 6.0, 50 mm pyruvate, 200 mm KHCO_3_, 0.5 mm MnCl_2_, and 0.6 mm NAD(P)H.

### Cultivation of human THP-1 macrophages

The THP-1 human monocytic cell line was obtained from ATCC TIB-202. Cells were grown in RPMI 1640 medium supplemented with 0.2% glucose, 0.2% sodium bicarbonate, and 10% heat inactivated fetal calf serum (FCS) (Sigma).

### Intracellular bacterial growth assays

THP-1 cells were differentiated with 50 nm phorbol 12-myristate 13-acetate for 72 h at 37 °C, 5% CO_2_, and 95% humidity. Cells were washed with PBS supplemented with 0.49 mm Mg^2^ and 0.68 mm Ca^2+^ (PBS+) and 1% FCS. Macrophages were infected with different strains of *Mtb* at a multiplicity of infection of 0.1 for 4 h at 37 °C. Extracellular bacteria were removed by washing twice with warm PBS containing 1% FCS. Intracellular bacteria were quantified by lysing the cells with 0.1% Triton X-100 at the indicated time points and plating dilutions on 7H11 agar.

### ^13^C isotopologue profiling of intracellular Mtb

^13^C isotopologue profiling was performed as described previously (15). Briefly labeled or unlabeled THP-1 macrophages were infected with different strains of *Mtb* at a multiplicity of infection of 5. After a 4-h incubation the macrophages were washed and incubated for 48 h before the mammalian and bacterial fractions were separated and used as a probe for the analysis of ^13^C labeling of amino acids in host macrophages and intracellular *Mtb* respectively.

### ^13^C biomass hydrolysate and preparation of amino acid derivatives

Amino acid derivatives were prepared from bacterial and host cell fractions as previously described (15). These were corrected for the natural abundance of all stable isotopes.

### Extraction and analysis of lipids from mycobacterial strains

Extraction of polar, apolar lipids, and MAMEs from the conditional mutant, and subsequent TLC analysis was carried out using protocols described in Ref. 18. Dry weights of cell pellets were used as a measure to equalize loading on TLC plates using solvent systems A–D for apolar lipids and systems D and E for polar lipids. The GC-free induction decay analysis of FAMEs was performed on a Shimadzu GC-2010. The FAMEs separation was achieved using a DB-225 column (30 m × 0.25-mm inner diameter, 0.25 μm film thickness, J&W Scientific) with a column flow of 1.26 ml/min (helium at a constant pressure of 100 kilopascal). Injector and detector temperatures were 50 °C. The injection volume was 5 μl. The column temperature was programmed initially at 50 °C for 2 min, increased at the rate of 4 °C/min to 220 °C, and then maintained at 220 °C for a further 30.5 min. The total program time was 75 min.

## Author contributions

P. B., N. S., A. B., A. S., R. B., I. G., N. A. C., L. G., and D. J. B. investigation; A. B., T. A. M., J. L. W., M. B., and D. J. B. formal analysis; A. B. and D. J. B. methodology; A. B., T. A. M., J. M., and D. J. B. writing-review and editing; D. J. B. conceptualization; D. J. B. supervision; D. J. B. funding acquisition; D. J. B. writing-original draft; D. J. B. project administration.

## Supplementary Material

Supporting Information

## Footnotes

2 The abbreviations used are:

Mtb: *Mycobacterium tuberculosis*
TB: tuberculosis
PEP: phosphoenolpyruvate
^13^C-FSA: ^13^C-flux spectral analysis
OAA: oxaloacetate
ANA: anaplerotic
PCA: pyruvate carboxylase
PCK: PEP carboxykinase
MEZ: malic enzyme
PPDK: pyruvate phosphate dikinase
FA: fatty acid
FAME: fatty acid methyl ester
MAME: mycolic acid methyl esters
FAS: fatty acid synthase
PDH: pyruvate dehydrogenase
X-gal: 5-bromo-4-chloro-3-indolyl β-d-galactoside
FCS: fetal calf serum.
