# Supplementary material for: The anaplerotic node is essential for the intracellular survival of Mycobacterium tuberculosis
Source: J Biol Chem. 2018 Feb 23;293(15):5695–704. doi: 10.1074/jbc.RA118.001839 (PMC5900758; doi:10.1074/jbc.RA118.001839)
Supplement: Supporting Information [file supp_293_15_5695__index.html]

The anaplerotic node is essential for the intracellular survival of Mycobacterium tuberculosis — The anaplerotic node of Mtb — The anaplerotic node is essential for the intracellular survival of Mycobacterium tuberculosis — The anaplerotic node of Mtb — Supporting Information 

# The anaplerotic node is essential for the intracellular survival of *Mycobacterium tuberculosis*

## Supporting Information

- Supporting information - 13C isotopologue data, TLC results and mutant growth data
