# Supplementary material for: The anaplerotic node is essential for the intracellular survival of Mycobacterium tuberculosis
Source: J Biol Chem. 2018 Feb 23;293(15):5695–704. doi: 10.1074/jbc.RA118.001839 (PMC5900758; doi:10.1074/jbc.RA118.001839)
Supplement: Supporting Information [file supp_RA118.001839_135180_2_supp_78573_p432b6.pdf]

Supporting Information

The essential role of the anaplerotic node in the intracellular survival of *Mycobacterium tuberculosis*

Piyali Basu[1], Noor Sandhu[1], A. Bhatt[2], A. Singh[2], Ricardo Balhana[1], Irene Gobe[1], Nicola A Crowhurst[1], Tom A Mendum[1], Liang Gao[3], Jane L Ward[3], Michael H Beale[3], Johnjoe McFadden[1] and Dany JV Beste[1]*

Table S1. Sole carbon sources utilised by wild type and mutant strains of *Mycobacterium tuberculosis*

		**Strain**									
**Carbon source**	**WT**	**Δ*mez***	**Δ*pca***	**Δ*pca*: *pca***	**Δ*pck***	**Δ*pck*: *pck***	**Δ*ppdk***	**Δ*ppdk*: *ppdk***	**Δ*pca* - Δ*pck***	**Δ*pca* Δ*pck:pck***	**Δpca Δpck:pca**
Ala (O_2)	+	+	NG	++	NG	+++	++	+++	NG	NG	NG
Ala (CO_2)	++	NT	++	++	NG	+++	+++	+++	NG	+++	NG
Ace (O_2)	+++	+++	+	NT	NG	+++	+++	NT	NG	+++	NG
Ace (CO_2)	+++	NT	++	NT	NG	+++	+++	+++	NG	+++	NG
Chol (O_2)	+++	+++	+	NT	NG	++	NG	+++	NG	+++	NG
Chol (CO_2)	+++	NT	+	NT	NG	++	NG	+++	NG	+++	NG
Gluc (O_2)	+	+	+	NT	+	NT	+	NT	+	NT	NT
Gluc (CO_2)	+	NT	+	NT	+	NT	++	NT	+	NT	NT
Glyc (O_2)	++	++	++	++	++	++	+	NT	NG	NG	++
Glyc (CO_2)	+	NT	+	++	+++	++	++	++	+++	+++	+++
Glu (O_2)	+	+	+	NT	NG	+	+	+	NG	+	NG
Glu (CO_2)	+	NT	+	NT	NG	+	+	+	NG	+	NG
Pyr (O_2)	++	++	NG	+	NG	++	++	++	NG	NG	NG
Pyr (CO_2)	++	NT	+	+	NG	++	++	++	NG	+++	NG
Ser (O_2)	+	+	NG	+	NG	+	++	++	NG	NG	NG
Ser (CO_2)	+	NT	+	NT	NG	++	++	+	+	+	NG
Succ (O_2)	++	++	+	NT	NG	+++	+++	+++	NG	+++	NG
Succ (CO_2)	++	NT	+	NT	NG	+++	+++	+++	NG	+++	NG

Ala: alanine; Ace: acetate; Chol: cholesterol; Gluc; glucose; Glyc: glycerol; Glu: glutamate; Pyr: pyruvate; Ser: serine; Succ: succinate; O_2: ambient air; CO_2: 5 % CO_2; NG: no growth; NT: not tested; Growth of 20 µl spots from: +: neat; ++: 10^{-1}; +++: 10^{-2} dilutions.

Table S2. Multiple carbon sources utilised by wild type and mutant strains of *Mycobacterium tuberculosis*, related to Fig. 5

**Carbon sources**	**WT**	**Δ*mez***	**Δ*pca***	**Δ*pca:pca***	**Δ*pck***	**Δ*pck:pck***	**Δ*ppdk***	**Δ*ppdk:ppdk***	**Δ*pca* Δ*pck***	**Δ*pca* Δ*pck:pck***	**Δ*pca* Δ*pck:pca***
Ala/Asp (O_2)	+	+	+	NT	NG	+	+	NT	NG	+	NT
Ala/Asp (CO_2)	+	NT	+	NT	NG	+	NT	NT	NG	+	NT
Ace/chol (O_2)	+++	+++	NT	NT	NG	+++	NG	+++	NG	+++	NT
Ace/chol (CO_2)	+++	NT	NT	NT	NG	++	NG	+++	NG	+++	NT
Pyr/chol (O_2)	++	++	NT	NT	NG	++	NG	+++	NG	NG	NG
Pyr/chol (CO_2)	++	NT	NT	NT	NG	++	NG	+++	NG	++	++
Glyc/chol (O_2)	+	+	NT	NT	+	+	NG	+	++	+	++
Glyc/chol (CO_2)	+	NT	NT	NT	+	+	NG	+	++	+	++
Glu/chol (O_2)	++	++	NT	NT	NG	++	NG	++	NG	++	NG
Glu/chol (CO_2)	+	NT	NT	NT	NG	++	NG	+	NG	+	NG
Ace/pyr (O_2)	+++	+++	+++	+++	NG	++	++	+++	NG	+++	NT
Ace/pyr (CO_2)	+++	NT	+++	+++	NG	+++	+++	+++	NG	+++	NT
Pyr/Asp (O_2)	+	+	+	NT	NG	++	+	NT	NG	++	NT
Pyr/Asp (CO_2)	+	NT	+	NT	NG	++	+	NT	NG	++	NT
Succ/Pyr (O_2)	++	++	+++	+++	NG	++	++	++	NG	++	NT
Succ/Pyr (CO_2)	++	NT	+++	++	NG	++	+	++	NG	+++	NT
Glu/ala/ser/asp (O_2)	++	++	++	++	NG	++	++	++	NG	+++	NT
Glu/ala/ser/asp (CO_2)	++	NT	++	++	NG	++	++	++	NG	+++	NT
Ace/Asp/Glu/Ala/pyr (O_2)	++	++	++	++	NG	++	++	++	NG	++	NT
Ace/Asp/Glu/Ala/pyr (CO_2)	++	NT	+++	++	NG	++++	+++	+++	NG	+++	NT

Ala: alanine; Ace: acetate; Asp: aspartate; Chol: cholesterol; Glyc: glycerol; Glu: glutamate; Pyr: pyruvate; Ser: serine; Succ: succinate; O2: ambient air; CO_2: 5 % CO2; NG: no growth; NT: not tested; Growth on 20 µl spots from: +: neat; ++: 10^{-1}; +++: 10^{-2} dilutions.

Table S3. Total ^{13}C (%) incorporation of proteogenic amino acid derivatives of intracellular *Mtb, Δpca and* Δ*pca* Δ*pck* cultivated with sodium [^{13}C]bicarbonate						
	**WT H37Rv**		**Δ*pca***		**Δ*pca* Δ*pck***	
**AA**	**Average (%)**	**Stdev**	**Average (%)**	**Stdev**	**Average (%)**	**Stdev**
**Ala**	1.68	0.26	0.28	0.16	0.10	0.11
**Gly**	3.86	0.63	1.25	0.11	0.64	0.86
**Val**	1.37	0.15	0.08	0.12	0.00	0.00
**Leu**	0.16	0.26	0.00	0.00	0.00	0.00
**Threo**	7.46	1.60	1.84	0.08	0.20	0.17
**Asp**	7.57	0.65	3.32	0.39	2.42	1.21
**Met**	5.69	1.88	1.39	0.27	0.88	0.45
**Phe**	2.65	1.44	0.26	0.36	0.00	0.00
**His**	2.74	1.10	0.61	0.03	0.00	0.00
**Tyr**	5.00	0.94	0.95	1.34	0.01	0.02

Table S4. Experimental (GC-MS) mass distributions in the amino acid derivatives of intracellular wild type H37Rv Mtb and Δppdk

		WT *Mtb* H37Rv		Δ*ppdk*	
**AA**	**Analyte (m/z)**	**Average**	**Stdev**	**Average**	**Stdev**
**Ala260**	**M+0**	**84.91**	**5.12**	**78.97**	**1.70**
	**M+1**	**2.81**	**0.46**	**0.98**	**0.13**
	**M+2**	**1.92**	**0.38**	**1.57**	**0.12**
	**M+3**	**10.35**	**5.33**	**18.48**	**1.54**
**Gly246**	**M+0**	**94.19**	**1.18**	**97.18**	**0.11**
	**M+1**	**1.81**	**0.75**	**0.00**	**0.00**
	**M+2**	**4.00**	**1.21**	**2.82**	**0.11**
**Val288**	**M+0**	**92.49**	**1.40**	**98.76**	**0.17**
	**M+1**	**4.78**	**1.26**	**0.40**	**0.20**
	**M+2**	**1.81**	**0.35**	**0.39**	**0.13**
	**M+3**	**0.85**	**0.21**	**0.26**	**0.05**
	**M+4**	**0.15**	**0.03**	**0.17**	**0.07**
	**M+5**	**<LOQ**	**<LOQ**	**0.02**	**0.01**
**Leu302**	**M+0**	**94.71**	**1.73**	**97.64**	**1.54**
	**M+1**	**2.07**	**0.69**	**2.33**	**1.42**

	**M+2**	**2.51**	**0.86**	**0.42**	**0.27**
	**M+3**	**0.44**	**0.25**	**0.25**	**0.23**
	**M+4**	**0.26**	**0.11**	**0.01**	**0.01**
	**M+5**	**0.05**	**0.03**	**0.00**	**0.00**
	**M+6**	**<LOQ**	**<LOQ**	**0.05**	**0.03**
**Ser**$_{390}$	**M+0**	**86.27**	**2.54**	**97.83**	**0.56**
	**M+1**	**3.97**	**1.33**	**0.00**	**0.00**
	**M+2**	**2.49**	**0.72**	**0.00**	**0.00**
	**M+3**	**7.27**	**4.60**	**2.17**	**0.56**
**Asp**$_{418}$	**M+0**	**84.45**	**2.53**	**84.71**	**0.34**
	**M+1**	**4.79**	**0.44**	**3.14**	**0.06**
	**M+2**	**6.98**	**1.04**	**6.95**	**0.27**
	**M+3**	**2.65**	**1.18**	**3.64**	**0.15**
	**M+4**	**1.14**	**0.61**	**1.56**	**0.07**
**Glu**$_{432}$	**M+0**	**84.28**	**2.89**	**81.66**	**2.67**
	**M+1**	**4.35**	**1.12**	**4.52**	**0.66**
	**M+2**	**7.15**	**1.24**	**6.45**	**3.81**
	**M+3**	**1.94**	**0.57**	**3.38**	**0.81**
	**M+4**	**1.50**	**0.76**	**2.89**	**0.51**
	**M+5**	**0.77**	**0.44**	**1.11**	**0.51**

**Phe$_{336}$**	**M+0**	**92.08**	**3.03**	**98.97**	**0.32**
	**M+1**	**3.70**	**1.96**	**0.00**	**0.00**
	**M+2**	**2.46**	**0.92**	**0.11**	**0.14**
	**M+3**	**1.25**	**0.54**	**0.17**	**0.05**
	**M+4**	**0.69**	**0.30**	**0.15**	**0.10**
	**M+5**	**<LOQ**	**<LOQ**	**0.08**	**0.01**
	**M+6**	**<LOQ**	**<LOQ**	**0.19**	**0.13**
	**M+7**	**<LOQ**	**<LOQ**	**0.10**	**0.03**
	**M+8**	**<LOQ**	**<LOQ**	**0.29**	**0.28**
	**M+9**	**<LOQ**	**<LOQ**	**0.09**	**0.07**
**His$_{440}$**	**M+0**	**86.81**	**5.09**	**97.86**	**0.09**
	**M+1**	**7.42**	**3.26**	**0.05**	**0.08**
	**M+2**	**3.39**	**1.20**	**0.47**	**0.16**
	**M+3**	**1.80**	**0.64**	**0.98**	**0.13**
	**M+4**	**0.44**	**0.20**	**0.28**	**0.11**
	**M+5**	**0.19**	**0.17**	**0.30**	**0.09**
	**M+6**	**<LOQ**		**0.05**	**0.04**
**Tyr$_{376}$**	**M+0**	**83.16**	**6.54**	**95.95**	**0.52**
	**M+1**	**9.18**	**4.38**	**0.14**	**0.12**
	**M+2**	**4.08**	**1.65**	**0.44**	**0.08**

	**M+3**	**2.20**	**1.15**	**1.51**	**0.13**
	**M+4**	**1.07**	**0.69**	**0.23**	**0.10**
	**M+5**	**0.41**	**0.18**	**0.32**	**0.14**
	**M+6**	**<LOQ**	**<LOQ**	**0.63**	**0.20**
	**M+7**	**<LOQ**	**<LOQ**	**0.40**	**0.42**
	**M+8**	**<LOQ**	**<LOQ**	**0.23**	**0.12**
	**M+9**	**<LOQ**	**<LOQ**	**0.20**	**0.06**

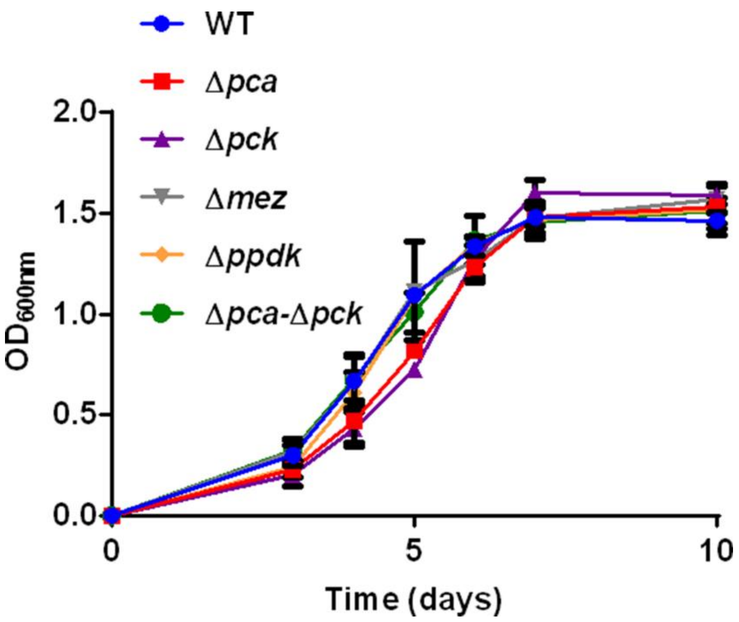

Figure S1. Deleting genes of the ANA node has no impact on growth in standard Middlebrook 7H9 medium

Growth (OD^{600}) of WT (blue), Δ*pca* (red), Δ*pck* (purple), Δ*ppdk* (yellow), Δ*mez* (grey) Δ*pca*-Δ*pck* (green) strains of *Mtb* in 7H9 media. The mean OD600 ± SEM of triplicate independent cultures is given.

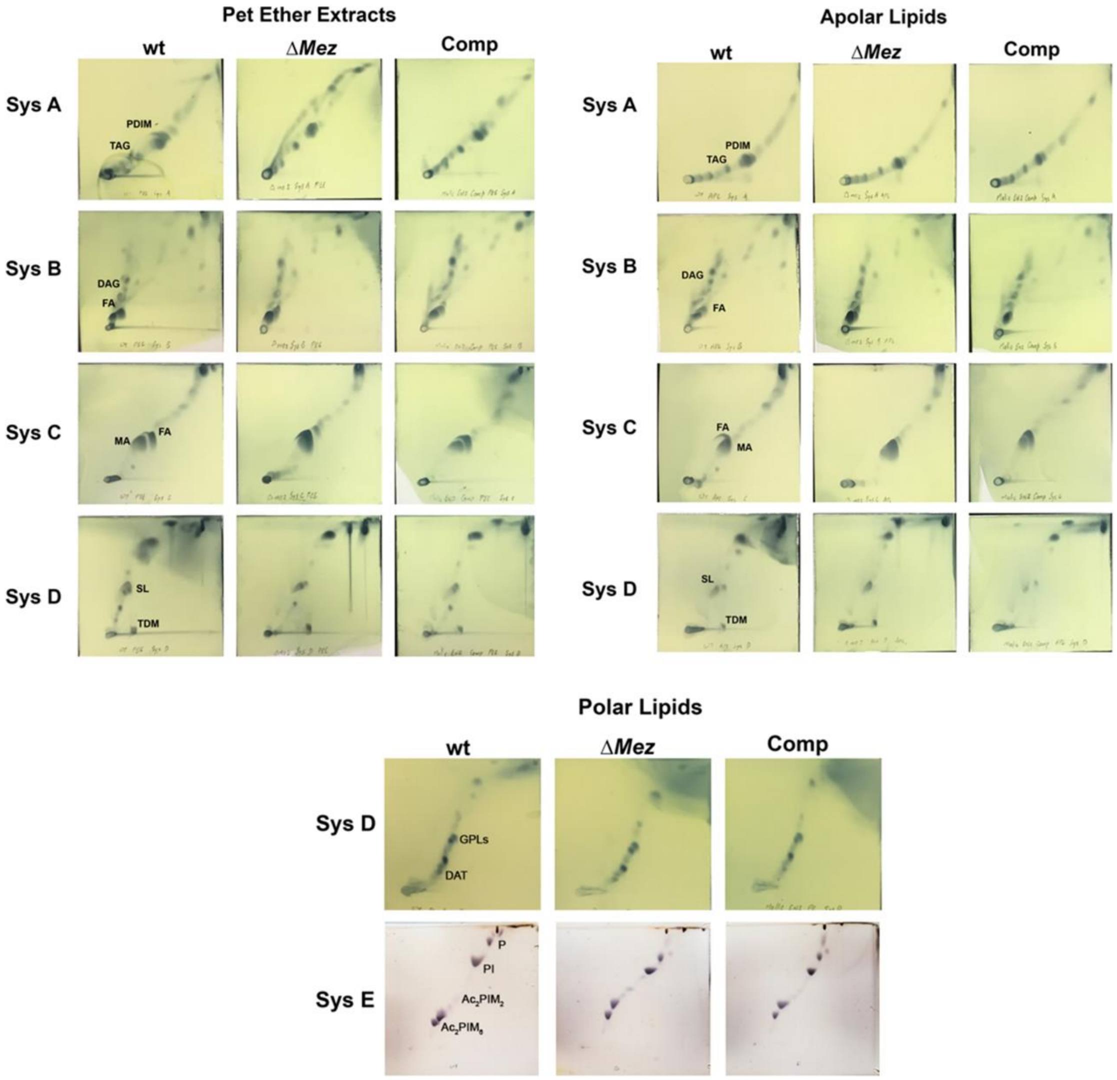

Figure S2. 2D-TLC Analysis

Analysis of petroleum ether extracts, and subsequently extracted apolar and polar lipids from WT, *Δmez* and *Δmez: mez.* Solvent systems A-E are as described by Dobson *et al* (1985).

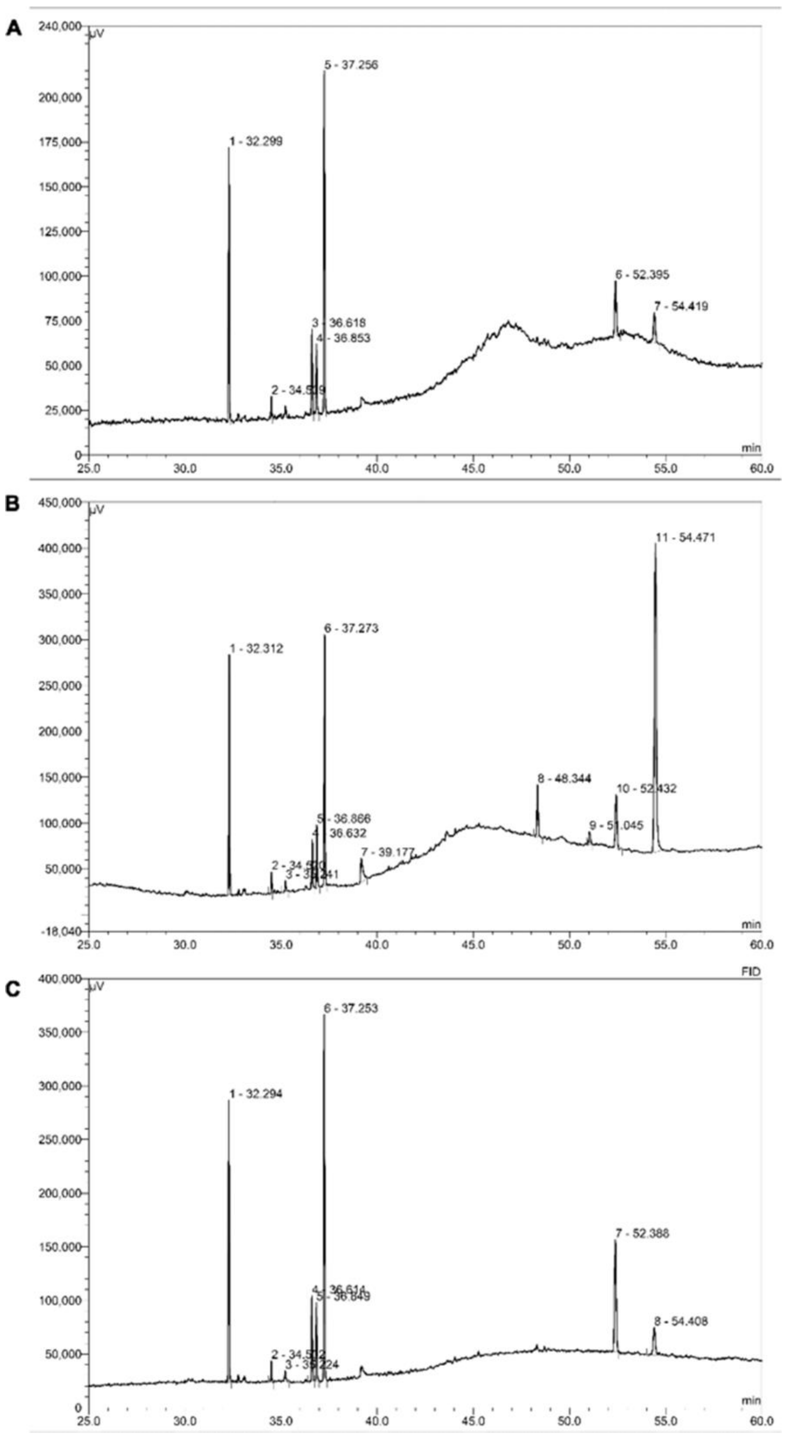

Figure S3. GC-MS analysis of fatty acyl methyl esters prepared from apolar lipids shows an increase of a C26 lipid species at 54.5 in Δ*mez*

GC-MS of WT (A), Δ*mez* (B) and complemented *Δmez: mez* strains of *Mtb*. Sigma-Aldrich fatty acyl methyl esters were used as standards.
